# Maritime Data Transfer Protocol (MDTP): A Proposal for a Data Transmission Protocol in Resource-Constrained Underwater Environments Involving Cyber-Physical Systems

**DOI:** 10.3390/s17061330

**Published:** 2017-06-08

**Authors:** Jesús Rodríguez-Molina, Belén Martínez, Sonia Bilbao, Tamara Martín-Wanton

**Affiliations:** 1 Research Center on Software Technologies and Multimedia Systems for Sustainability (Centro de Investigación en Tecnologías Software y Sistemas Multimedia Para la Sostenibilidad—CITSEM), Campus Sur UPM, Ctra. Valencia, Km 7, 28031 Madrid, Spain; 2 Tecnalia Research & Innovation, Parque Científico y Tecnológico de Bizkaia, C/Geldo, Edificio 700, 48160 Derio, Spain; belen.martinez@tecnalia.com (B.M.); sonia.bilbao@tecnalia.com (S.B.); 3 HI Iberia Ingeniería y Proyectos, Juan Hurtado de Mendoza 14, 28036 Madrid, Spain; tmartin@hi-iberia.es

**Keywords:** autonomous maritime vehicles, protocol, protocol data unit

## Abstract

The utilization of autonomous maritime vehicles is becoming widespread in operations that are deemed too hazardous for humans to be directly involved in them. One of the ways to increase the productivity of the tools used during missions is the deployment of several vehicles with the same objective regarding data collection and transfer, both for the benefit of human staff and policy makers. However, the interchange of data in such an environment poses major challenges, such as a low bandwidth and the unreliability of the environment where transmissions take place. Furthermore, the relevant information that must be sent, as well as the exact size that will allow understanding it, is usually not clearly established, as standardization works are scarce in this domain. Under these conditions, establishing a way to interchange information at the data level among autonomous maritime vehicles becomes of critical importance since the needed information, along with the size of the transferred data, will have to be defined. This manuscript puts forward the Maritime Data Transfer Protocol, (MDTP) a way to interchange standardized pieces of information at the data level for maritime autonomous maritime vehicles, as well as the procedures that are required for information interchange.

## 1. Introduction

Cyber-Physical Systems (CPSs) are regarded as most useful due to the fact that they combine the advantages of distributed systems, hardware developments and networked information. One of the main issues that these developments present is the transmission of data between the different entities taking part in the communications. Even though there is a collection of technologies proven to be fully functional in regular environments, having a suitable protocol to interchange information among nodes becomes way more challenging when constrained environments, such as underwater and subsea environments, are providing the transmission medium.

### 1.1. Cooperating Autonomous Maritime Vehicles as a Cyber-Physical System

There are several ways to describe CPSs according to the existing literature. For Rajkumar et al. [1], they are physical and engineered systems that operate in a way that their elements are controlled, monitored, coordinated and integrated by an underlying core of computing and communication operations. According to [2], they imply a tight integration of physical, communication and computation elements ranging from medical devices to transportation and energy systems. All in all, although CPSs can be described in many different ways, they usually imply a collection of devices distributed in a certain location that interact with the environment by means of software-based commands that are sent and received with a minimal interaction from a human end user. This is no exception regarding the integration of communications at the data level regarding maritime robotics. This latter kind of devices can be better described as the usual vehicles manufactured to perform over- and subsea operations. Several examples of them are: Autonomous Underwater Vehicles (AUVs), Autonomous Surface Vehicles (ASVs), Unmanned Surface Vehicles (USVs) or a Remote Operated Vehicles (ROVs). Some examples of the operations they will be involved in are navigation in unknown underwater environments with simultaneous data gathering [3], river tracking and navigation while observing as much as possible The International Regulations for Preventing Collisions at Sea (COLREGs) [4], as components of distribution management systems with aerial vehicles [5], and as hardware components for underwater sensors networks [6].

Having a myriad of these vehicles working cooperatively under a common mission for all of them can be regarded as a CPS, where several physical elements are integrated in a deployment where vehicles gather data and are controlled and/or monitored by an entity responsible for taking decisions or designing the policies of that system. Unfortunately, a CPS with those features also presents important challenges that must be addressed, both related to the maritime environment (low bandwidth for data transmissions, unreliable channel for information exchanges, environment with a high degree of unpredictable features such as seawater composition, underwater currents, etc.) and to the distributed nature of the system (communications among heterogeneous hardware elements deployed in a certain environment). Among other pieces of work, these challenges are stated in [7], where it is mentioned how the movement added to the underwater nodes by submarine currents makes underwater routing very unreliable, and [8], where it is explicitly claimed that “*Underwater acoustic communications are notoriously prone to interferences, disruption and unpredictable delays. Furthermore, the available bandwidth is usually very limited and the latency is large*”.

As in any other CPS, information must be transferred among the elements used to collect information and interact with the application domain where those devices are included. This is a mandatory feature for the kind of environment that is described in this manuscript because there are several use cases where different types of information must be exchanged:

1. If an infrastructure that requires pillars or columns has been installed in the sea (for example, an offshore windmill or an oil platform), maintenance operations can make use of a CPS composed by several AUVs to monitor those pillars searching for cracks or other signs of structural fatigue in a cheaper—and less dangerous for human lives—manner than if there were human divers participating in the surveying operations.
2. In a similar way, should there be a need to explore any subsea, pipe-like structure (telecommunication cables, underwater pipelines) it can be done in a more efficient manner by deploying several underwater vehicles. In this way, they will be able to perform monitoring activities on the pipe and share the information obtained from the vehicles in a way that services can be composed from the data acquired.
3. Another practical example would imply using a set of underwater vehicles to supervise the building of a subsea berm: having several vehicles deployed at once will reduce the time required to scan all the subsea structure, and will allow a faster assessment of the construction works performed in that location.

### 1.2. The Challenging Nature of Communications in Maritime Environments

Communication is the most critical process in underwater technology. It can be established either by wired or wireless connections. Both methods have their own advantages and disadvantages, depending on the application. The current trend favors wireless communications as the most suitable way to transfer information, especially when it comes to deal with environments like the one described in this manuscript, where wired connections are either not practical or just impossible [9].

Unlike terrestrial-based applications, establishing underwater wireless communications is not a straightforward process. When considering the underwater communications, the main concerns that researchers consider involve the channel model (in this case, underwater), attenuation, transmission distance, power consumption, Signal-to-Noise ratio, bit error, symbol interference, error coding, modulation strategies, instrumentation and underwater interferences. Dealing with interferences for underwater research is a complex task due to the dynamic nature of water, which makes it difficult to plan communication links ahead.

As far as AUVs are concerned, issues of navigation and communications are often the most difficult problems to address, as there are few options for transmitting messages underwater [2]. Unlike radio links in terrestrial applications, challenges are quite different for underwater wave propagation. Water itself becomes the main source for the signal interference, due to unpredictable changes in some of the parameters that define most prominent features of seawater, like permittivity [10]. Indeed, characteristics like water turbidity, temperature and water noise have an impact in acoustic wave propagation [11]. In addition to that, common terrestrial phenomena like scattering, reflection or refraction also happens in underwater application domains [12]. There are various ways to get reflection in underwater environment, for example the signal can bounce off the sea floor and other underwater geographic structures, including softer mediums such as the ocean surface and layers of water separated by differences in temperature or density.

In the underwater area of knowledge there are three types of carrier waves that are most commonly used in wireless communications: (a) electromagnetic waves, (b) optical waves and (c) acoustic waves [13,14]. Using electromagnetic waves, the communication can be established at a higher frequency and bandwidth, but there are limitations due to high absorption/attenuation, which has a significant effect on the transmitted signal. A large antenna is also needed for this type of communication, thus affecting the design complexity and cost. Optical waves also offer high data rate transmission. Unfortunately, the signal is rapidly absorbed in water and suffers from scattering effect [3,4,5,6], which affects data transmission accuracy. Acoustic is the most preferred signal used as carrier by many applications because of its low absorption characteristics for underwater communication. Even though the data transmission is slower compared to other carrier signal, low absorption features enable the carrier to travel at longer range as less absorption is faced by the carrier. Depending on the distance between endpoints of a data transmission, bandwidth can range from 2 kbits [15] to 87 kbits per second [16].

When ensuring effective underwater communication, the communication system design plays a vital role. Factors such as transducer parameters (sensitivity, power consumption, noise immunity, transduction mechanism, directivity, resolution) and properly matched impedance must be taken into account during the process. Also, from the instrumentation system point of view, the size of devices is one of the main concerns. Manufacturers from the electronics industry are competing to produce a device with better performance and smaller size for the overall efficient system. For example, even though AUVs are very different from one manufacturer to another one, they can range from 80 cm (Naiad, [17]) through 170 cm (Remus 100, [18]) to 2 m and 9 inches of diameter in case of ECA’s A9 [19]. Thus, it becomes clear that many practical use cases require data interchanges among vehicles deployed in a mission. Therefore, how information can be transferred from one underwater vehicle to another becomes a topic of major importance in CPS based on autonomous maritime vehicles. As it has been previously mentioned, there are several aspects that must be dealt with for the successful transmission of data: the unreliability of the underwater environment, how constrained the transmission medium is whenever any information has to be transmitted or the kind of information of interest for the CPS have to be considered. All these aspects must be apprehended by any protocol used for information exchanges at the data level for underwater environments. These characteristics are even more important for higher level of information transmissions, as there are challenges like the format of the data to be transmitted, or how those pieces of information are going to be included in the Protocol Data Units, that must be addressed by any protocol to be designed.

Overall, the challenges that must be faced by underwater communications at the data level can be summarized as follows:

1. *Scarce bandwidth*. Transmissions at the data level often rely on information formats that are more verbose than in other layers (XML files, SPARQL requests, JSON messages), so transmitting this kind of information in a distributed system poses a significant challenge when information is requested by any human handling a system where a significant amount of information has to be used.
2. *Low reliability*. The means of transmission used for underwater communications are usually not fully trustable, so protocols that tackle this issue to an extent in different ways (for example, by adding redundancy, having small PDUs, etc.). Even by using acoustic waves, which is regarded as the most effective way to transmit data underwater, reliability of the transmissions is significantly lower than in a regular wireless transmission.
3. *Varying conditions of the environment*. Due to the inherent conditions of any maritime deployment that is carried out in open waters, chemical characteristics of the medium (salt composition, water pollution, etc.) as well as mechanical ones (subsea currents, periodic tides, etc.) the conditions of the surroundings where a deployment is supposed to be done change frequently; this can be a challenge for any system that is required to be stable, as these conditions will vary greatly every day.

### 1.3. Paper Contributions and Structure

There are several unique contributions that this paper makes when compared to other works available in the literature that has been studied by the authors of this proposal:

1. A protocol that has been specifically defined for underwater environments is put forward. This has been done so by collecting information among the consortium members of the Smart and Networking Underwater Robots in Cooperation Meshes (SWARMs) research project [20], which have provided their feedback regarding the kind of information that must be transferred among the different components of a CPS with autonomous maritime vehicles.
2. After the design was completed and deemed satisfactory, an implementation of that protocol was carried out. This was done so as a way to test the reliability of the Protocol Data Units (PDUs) that were previously designed, as well as the possibility to develop an actual piece of work based on a regular programming language. Java has been used for such a purpose, although it was not the only software resource that has been utilized.
3. The underlying implementation works that were performed made use of Data Distribution Service (DDS) as a way to provide data interoperability among the different devices that can be integrated by the same protocol. While DDS facilities imply several software developments that have been already tested in constrained environments (such as a Data-Centric Publish/Subscribe—referred to as DCPS—or a Real-Time Publish Subscribe—RTPS—wire protocol used for interoperability among DDS solutions), the protocol that has been designed and implemented is completely new and, to the best of the authors’ knowledge, there is not a development like this in any other proposal with this kind of characteristics.

## 2. Related Works

The solutions that solve challenges resembling the ones described here have been considered.

### 2.1. A Deadline-Constrained 802.11 MAC Protocol

Tian et al. put forward in their proposal what is conceived as a way to handle periodic traffic expected to arrive within a timespan [21]. The authors put forward a deadline-constrained Medium Access Control (MAC)-based scheme with Quality of Service (QoS) features that relies on an intra-Access Categories (AC) with QoS differentiations to directly meet the deadlines imposed by real-time communications. On the other hand, a contention-sensitive Binary Exponential Backoff (BEB) algorithm has been added as a way to enhance backoff delay performance. According to the authors, Network Simulation version 2 has been the simulator of choice for the performance verification. There are two scenarios that have been prepared: one compares the performance of the chosen protocol with another defined by the IEEE 802.11a (Enhanced Distributed Control Access, EDCA) with regards to average delay and packet loss ratio; these tests prove that the MAC scheme conceived by the authors behaves better than EDCA for constrained environments. The second scenario makes use of 20 nodes gathered in two different kinds of groups with the same protocols as before; it is shown that when the period of time used to measure a critical period of time falls below 17 ms, the proposal fares better than EDCA, showing just a small delay and small packet losses.

As it will be seen in other proposals, the main issue is that this piece of work solves a problem located at a lower level from a layered architecture point of view; 802.11 and MAC protocols are located below the ones where data are transmitted as the fundamental PDU, so unfortunately, this solution is not applicable for the problem that is put forward in this manuscript.

### 2.2. Constrained Application Protocol (CoAP)

This solution aims to create a generic web protocol that can be used in constrained environments [22]. It partially relies on Representational State Transfer (REST) software style, an architectural style developed by Fielding [23]). The message format provided has also some prominent characteristics: to begin with, it offers a 4 byte header where several pieces of information (an identifier for the message, a code, version number of CoAP, etc.) have been included. Secondly, a token value is included right after the header of the message. Lastly, the payload encases the bulk of the message that is transmitted. Data transmissions usually involve an upper layer used to handle the queries and the responses sent from and to the applications; messaging is done to interact with lower levels of communications.

All in all, this proposal offers several advantages that must be taken into account for any solution used to transfer information at the data level for a specific environment, such as the PDU definition, interchange and purposes. The main disadvantage of this development is that it is expected to be used in environments that, while involving constrained resources, results more reliable that underwater communications. In addition to that, it is likely to work with data received from a regular transport layer, rather than the kind of frames received from acoustic modems, so its applicability for this kind of application domain is limited. If compared to the solution presented in this manuscript, the header of a CoAP message is still longer than the one in MDTP, so we manage to make a better use of the resources available.

### 2.3. Advanced Message Queuing Protocol (AMQP)

Advanced Message Queuing Protocol (AMQP) is an open wire Internet protocol for business messaging comprised of several layers [24]. The one located at the lowest level is used to interchange binary information on a peer-to-peer basis, whereas the immediately higher one is used to send and receive messages by using an abstract message format with specific standardized encoding. A higher level layer is used to contain a peer-to-peer transport-like protocol and the highest one makes use of either Transport Layer Security (TLS) or Simple Authentication and Security Layer (SASL) for security purposes such as encryption and authentication. The messaging level makes use of a messaging format, and another one is defined for transactions at a higher level. AMQP works in a way that it makes use of a communications broker to process all the requests that are performed. The broker provides communications under a Publish/Subscribe paradigm: publishers will send topics to the broker and they get organized in queues that are accessed by subscribers. There are several broker implementations that have been developed, such as Apache Qpid [25] or RabbitMQ [26].

AMQP offers a very detailed set of PDUs for information transmission at the data level (rather than being bits or packages). The main disadvantage of AMQP is that challenges like low bandwidth or the unreliability of the transmission medium have not been specifically taken into account, so it is not an advisable option to use in the context of this manuscript. Adding security at this level of data transmission can also be a major challenge, since encrypting data tends to result in rather verbose communications. Lastly, AMQP relies on some communication and transport protocols (such as TCP) that cannot be guaranteed to be available in an underwater level.

### 2.4. Multiple Access with Collision Avoidance (MACA)-Based Power Control

Qian et al. put forward their idea of a MAC protocol for Underwater Wireless Sensor Networks (UWSN) [27]. The authors describe a layered infrastructure where a physical, MAC/link, route, transmission and application layers are suggested as the levels present in such an infrastructure. The authors establish a classification to distinguish two different kinds of protocols (contention-based and contention-free). One kind of contention-based MAC protocol that uses handshaking is MACA, even though it is not adapted by default to underwater communications since it was not designed for such an environment. Thus, the authors show a MACA-based power control protocol (referred to as MACA-PC) in an underwater scenario with all the expectable interferences. Three concepts have been defined: the *transmission rage* (fixes the range at which packages can be received and decoded), the *interference range* (range at which nodes are capable of sending sender transmissions and can be interfered) and the *interference zone* (range where nodes from a UWSN can sense a transmission but cannot decode it). It was found out that MACA-PC offered a similar performance for a one-hop scenario as the output provided by regular MACA (and better than the one providing just basic power control, which is prone to have data collisions during transmissions) with a lower power consumption. A multi-hop scenario presented the same results.

The solution that is presented by the authors of the paper solves many of the issues that are found in underwater communications, but it cannot be applied to the application domain of this manuscript, due to the fact that the ideas that are put forward here involve interoperability at a higher level, where actual data rather than bits are transferred.

### 2.5. A power-Efficient Routing Protocol for Underwater Wireless Sensor Networks

Huang et al. put forward a protocol conceived to route information in the least energetically costly possible way [28]. They suggest that a combination between acoustic communications and radio links can be offered in order to offer a holistic solution for networked communications in a deployment. The architecture that has been designed for the power efficient routing protocol consists of several elements: firstly, it makes use of a *forwarding node selector*, which receives as an input transmission distances, the angle between the nodes of the network (it has to be taken into account that this is a 3D network), and the remaining energy of the sensor itself. All these data are sent to a *forwarding tree mining mechanism* that forwards the information as a packet that is transmitted to the sensor that was previously selected in the network. The other main purpose of this module is to prevent wasting power consumption due to fast spreading of packet forwarding procedures. Another development is the potential adoption of a fuzzy logic inference system, which would make use of fuzzing/defuzzing modules with an inference engine and a fuzzy rule base.

This solution puts forward an optimized scheme in order to transmit information among several entities that are contained in the application domain. The main issue with this presented solution is that it cannot be used for transmissions at the data level, since the protocol has been conceived for routing and other network layer functionalities. As it happened before, the usability of this proposal in the application domain described in this manuscript is reduced, because it deals with problems that are alien to the data transmissions done in any higher layer, and does not solve problems that may be found at this level.

### 2.6. Message Queue Telemetry Transport (MQTT)

MQTT is a messaging protocol that was introduced by Stanford-Clark of IBM and Nipper at Arcom (now Eurotech) in 1999 and standardized in 2013 by OASIS [29,30]. Each MQTT client can be either a publisher that sends information to the broker at a specific topic or/and a subscriber that receives automatic messages every time there is an update. MQTT utilizes the Transmission Control Protocol (TCP) to provide stable communications. Despite this, it is designed to have low overhead compared to other TCP-based application layer protocols [31]. To ensure security, MQTT brokers may require username and password authentication, which is handled by a Transport Layer Security (TLS) or Secure Sockets Layer (SSL); these are the same security protocols that ensure privacy for HTTP transactions all over the Internet [32].

These characteristics can be mapped with success to environments where the sensors are likely to be connected at least once in a while, and the lifecycle of the messages is managed by the protocol. However, messages delivered to the network may or may not be eventually received in this kind of environment, so an alternative using as a baseline for a more limited transport channel is needed. In addition to that, this solution relies on standardized protocols that may or may not be available during a mission involving underwater communications. Lastly, when compared to MQTT, CoAP is more lightweight-oriented, as it utilizes UDP as the transport protocol.

### 2.7. MQTT-SN (Message Queue Telemetry Transport for Sensor Networks)

MQTT-SN [33] is a variation of the MQTT protocol aimed at embedded devices on non-TCP/IP networks. The specifications provide three elements: connection semantics, routing, and endpoint. It is adapted to the peculiarities of a wireless communication environment such as low bandwidth, high link failures, short message length, etc. It is also optimized for the implementation on low-cost, battery-operated devices with limited processing and storage resources. MQTT-SN is characterized by the following differences: (a) the connection and messaging processes detail from the very outset of communication the topic that will be available for the Publish/Subscribe manager, (b) the topic name in the PUBLISH messages is replaced by a short, two-byte long “topic id”, (c) “Pre-defined” topic identifiers and “short” topic names are introduced, for which no registration is required, (d) a discovery procedure helps clients without a pre-configured server or gateway’s address to discover the actual network address of an operating server or gateway, (e) the semantic meaning of a “clean session” is extended to the Will feature (as stated in the connection process detailed above), i.e., not only client’s subscriptions are persistent, but also Will topic and Will message and (f) a new offline keep-alive procedure is defined for the support of sleeping clients.

All of these are useful characteristics in streamlining the process of Publish/Subscribe messaging, which is a must in bandwidth constrained environments, so messaging for a Publish/Subscribe paradigm has been taken into account while designing and implementing the main features of the proposed protocol.

### 2.8. Embedded Binary HTTP (EBHTTP)

Embedded binary HTTP (EBHTTP) [34] is a binary-formatted, space-efficient, stateless encoding of the standard HTTP/1.1 protocol. EBHTTP is primarily designed as the transportation of small scale data between resource-constrained nodes, which follows a similar approach to CoAP. This protocol is focused on reducing the overhead of HTTP while maintaining the same HTTP semantics and communication paradigm. EBHTTP uses the UDP protocol instead of TCP. The basic EBHTTP header consists of 2 bytes specifying the method and a control field; all other data are carried as Type-Length-Value (TLV) encoded sections of the method. These may include a request URI, HTTP headers (either compressed or uncompressed), and body data. This encoding also allows multiple EBHTTP messages to be packed into a single UDP datagram or TCP segment.

Some of the most important weaknesses relay on the lack of reliability and in the processing power required to encode the HTTP protocol. In fact, although the processing required to encode the protocol would be less than what is required by a direct use of HTTP, it would be still expensive due to the limited resources of a sensor. Furthermore, although they are bound on UDP, they do not provide any reliability mechanism. Lastly, it is a protocol that has been conceived for the transmission of information at the application layer, so its applicability for lower ones would be ineffective or plainly impossible to perform.

### 2.9. Extensible Messaging and Presence Protocol (XMPP)

The Extensible Messaging and Presence Protocol (XMPP) [35] is a message-oriented protocol for streaming eXtensible Markup Language (XML) elements with a real-time behavior. It is used combined with other very popular applications like Google App Engine [36]. Secure authentication (Simple Authentication and Security Layer or SASL) and encryption by means of Transport Layer Security (TLS) have been built into the core of XMPP specifications. XMPP runs over TCP and provides both Publish/Subscribe (asynchronous) and request/response (synchronous) messaging systems. It is designed for near real-time communications and thus, it supports small message footprint and low latency message exchange [37]. XMPP connects a client to a server using a stream of XML *stanzas*. An XML stanza represents a piece of code that is divided into three components: message, presence, and an iq pair (info/query). Message stanzas identify the source (from) and destination (to) addresses, types, and IDs of XMPP entities that retrieve data.

XMPP has TLS/SSL security built in the core of the specification. However, it does not provide QoS options that make it impractical for M2M communications, although XMPP supports the Publish/Subscribe architecture that is more suitable for the IoT in contrast to CoAP request/response approach. Furthermore, it is an already established protocol that is supported all over the Internet as a plus when compared to the relatively new MQTT. However, XMPP uses eXtensible Markup Language (XML) messages that create additional overhead due to unnecessary tags and require XML parsing.

### 2.10. Lightweight Machine-to-Machine Protocol

OMA Lightweight M2M (LWM2M) [38] is a protocol from the Open Mobile Alliance for M2M or IoT device management. It is a communication protocol used between client software on a M2M device and server software on a M2M management and service enablement platform. In order to use the LWM2M protocol for remote management of M2M facilities it has four characteristics: (1) it features an architectural design based on REST, which is usually appealing to software developers, (2) defines a resource and data model that is extensible, (3) has been designed with performance and the constraints of M2M devices in mind, and (4) it reuses and builds over an efficient secure data transfer standard, since CoAP has been standardized by the Internet Engineering Taskforce (IETF) as a variation of the Internet’s HTTP protocol (appropriate for data transfer to and from low-cost connected IoT devices).

As it can be inferred, LWM2M provides several interfaces built on top of Constrained Application Protocol (CoAP) to perform management of a wide range of remote embedded devices and connected appliances in the Internet of Things application domain, with the objective of performing remote service enablement and remote application management. LWM2M is targeted in particular at constrained devices, e.g., devices with low-power microcontrollers (40 MHz), small amounts of Flash (100 KB) and RAM (10 KB) or batteries expected to last for years over network variable availability [39].

### 2.11. Open Issues and Challenges

The solutions that have been described here show that interconnectivity challenges can be solved with success in environments where experience and knowledge have already been accumulated and there are technological solutions that can be applied to them. However, there is still as significant number of open issues and challenges that remain to be solved at mainly two different areas:

1. *Lack of usability in the environment of the application domain*. The solutions that have been described previously work successfully in environments that, despite being constrained or present important issues regarding computational capabilities, are usually no underwater environments or imply autonomous maritime vehicles. Therefore, its optimization to the application domain represented in the manuscript is likely to be suboptimal. What is more, there seems not to be efforts to port those developments to this environment, as they were never targeted to be used for autonomous maritime vehicles.
2. *Lack of developments for data level transmissions*. The solutions that have been described either focus on the transmission of information in underwater conditions at a different (and usually lower) layer, or when data level transmissions are taken into account, they are done at the application layer, rather than having a software development for a distributed system that will contain the information related to session and presentation levels.
3. *Lack of data about what should be part of the information that is transferred from/to the entities that take part in a communication*. Whereas there is a high consensus about the different kinds of information transferred in other constrained environments, such as Wireless Sensor Networks or the Internet of Things, the establishment of accurate criteria that define the specific data that have to be used in underwater transmissions (coordinates, vehicle speed, water temperature, composition, etc.) is yet to be defined.
4. *Lack of security options*. The security infrastructure offered by the studied proposals is often precarious or nonexistent. Usually, not even the option to send messages encrypted is available, which poses a threat to the whole system, that might not be secure enough to have it used. A way to guarantee secure data interchanges between ends of the communications must be guaranteed by any new protocol designed for the purpose of interchanging information at the data level in underwater environments.

The main advantages and disadvantages that have been found in each of them, as well as the references where the bulk of the information has been gathered, have been summarized in Table 1.

## 3. Description of the Maritime Data Transfer Protocol

The proposal that has been designed for data transfer in autonomous maritime vehicles is aimed to address all the open issues previously described by means of the following features:

1. The information that has been included is relevant and consistent with the needs of the partners and companies that are involved in the SWARMs project. Considering that the list of participants in the project ranges from autonomous maritime vehicle vendors to underwater acoustic modem manufacturers, it is believed by the authors of this manuscript that it fits quite accurately the requirements for information interchange. Specifically, feedback received from the network layer has been pivotal in order to tackle the issue of a constrained, unreliable transmission medium.
2. Data level transmissions. Unlike other protocols or decentralized data transmission proposals, information exchange has been conceived to be performed at the data level, so that it will be fully and easily ported to the autonomous maritime vehicles that are used in the project.
3. Underwater adaptation. The protocol has been designed from scratch for data transmissions in a maritime (and more specifically, underwater) environment. Again, the feedback provided by the partners of the SWARMs project has been of major importance in order to include all the relevant data in the PDUs. Therefore, they have been tailored in terms of types and fields used to contain the parameters of interest in a distributed system or a CPS with a significant component of autonomous maritime vehicles.

If the previous proposals are taken into account, there are several actions that have been carried out when developing MDTP in order to solve the challenges that have been previously presented. These actions have been summarized in Table 2.

It is due to all these reasons that the protocol has been named *Maritime Data Transfer Protocol*. Aside from the actions that have been taken by the developers of this protocol, there are several other advantages that can be offered due to the tools that have been used, such as time and space decoupling (due to the usage of DDS for this functionality, which allows publishers to connect whenever they are able to do so in case they become momentarily out of the deployment, as it is likely to happen from time to time in submarine environments), automatic discovery of nodes, quality of service guaranteed by default or optional security support. All the procedures undertaken that justify the PDUs design have been included.

### 3.1. Modelling Considerations

**Figure 1:**
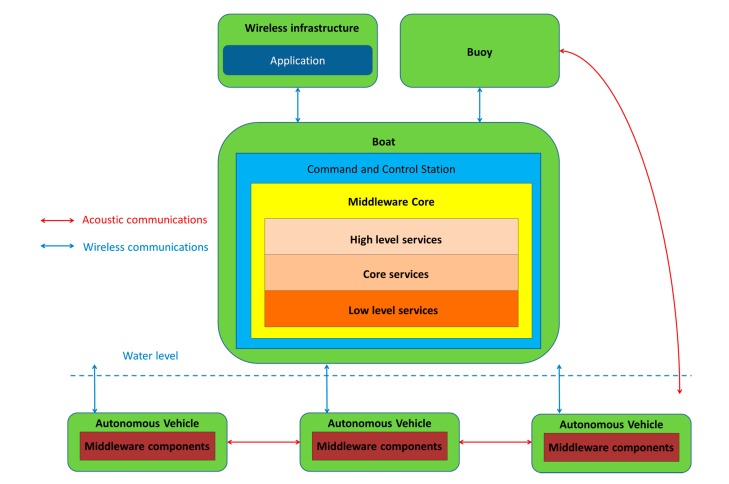
Distribution of middleware. MDTP is used over the acoustic and wireless network.

When operating with all the other elements from a mission in open sea, the protocol will be used among all the elements scattered in a certain area. It must be mentioned that MDTP has been conceived in cooperation with a software architecture that will be used in order to transfer information from the autonomous maritime vehicles to the actual middleware. By middleware, it is meant a software layer that is deployed in distributed systems or CPSs with the aim of abstracting the underlying hardware heterogeneity and complexity and providing the higher, more application layer-based elements, with a collection of facilities that are usually accessed via Application Programming Interface. Also, middleware can be enhanced by encasing several services within itself, ranging from device registration to security or semantic capabilities [40]. A most important characteristic is that the communications are made possible by the interaction with the autonomous maritime vehicles performing operations with an element containing a larger proportion of software components of the distributed middleware architecture, which is located in what is referred to as the Command and Control Station (CCS), as it has been depicted in Figure 1. Here, it can be seen how the overall structure of the system has been conceived: on the one hand, the CCS contains the bulk of the middleware components and all the services that can be deemed as high level (due to the fact that they are the closest to the applications and are used to access them), at the core (which contains the most prominent functionalities) and low level (mostly focused on hardware abstraction). On the other hand, the autonomous maritime vehicles will have their own share of middleware components, used to adapt the heterogeneous hardware to the resulting common system. In order to transfer the information between each of the parties, though, a protocol must be used at the data level whenever transmissions are done either Over-the-Air with regular wireless communications or via underwater acoustic network. Interestingly enough, the SWARMs project aims at unifying both networks, so using different protocols for data transmission can be counterproductive whenever information is shared between the autonomous maritime vehicles and the CCS. It is those communications where MDTP is used. Therefore, the figure also shows where the most prominent elements of the deployment that has been conceived will be running, as different pieces of hardware are required to have software components installed, which will be communicating to each other by means of MDTP.

The PDUs that have been designed for this protocol will make use of data interchange. The particular ideas that were taken into account to design the protocol were as follows:

1. Information of the entity that started the communication must be sent. The system running in the SWARMs project relies on the interchange of information at the data level among two different entities: the autonomous maritime vehicles and the equipment where all the other middleware elements have been included, which is referred to as the CCS. Depending on whether the vehicle or the middleware are publishing information or are subscribed to receive it, different PDUs must be used and different data will be included. Note that using protocols to measure flow information using TCP/IP layered architectures, such as IPFIX [41], would not be useful in this environment, due to the fact that a significant amount of communications will not rely on a regular IP infrastructure.
2. Communication channel. The message can be transmitted either overwater or underwater, using an IP or an acoustic communication channel which highly influences the speed and amount of bytes that can be efficiently delivered from the sender to the receiver. The most evident consequence to deal with is that the PDUs that are more verbose will be sent via the IP network. Even though it cannot be cabled by any means, any network relying on already established standards for wireless communications will work in an easier way than at the acoustic level.
3. Periodicity of the message. There will be messages that will be sent periodically as in any other distributed system or CPS. Commonly, these ones will be related to periodic heartbeats or keep alive notifications used to communicate both the autonomous maritime vehicles with each other, along with the CCS.
4. Purpose of the message. Depending on the nature of the information contained in the message (data requests, GPS coordinates, etc.) the messages that are used will be interchanged depending on what has been requested and the availability of information.

Consequently, a taxonomy that classifies the messages that have been created according to the entity that sends them (that is, either the autonomous maritime vehicle or the CCS) has been created as a way to provide a better grasp of the messages that are used.

### 3.2. Taxonomy of Messages

The messages sent by a vehicle have been grouped under four categories:

1. Status information is published periodically and usually corresponds to proprioceptive data, i.e., data about the vehicle itself, such as its estimated position or speed.
2. A report is a collection of data about the vehicle or its environment (exteroceptive data) aggregated to answer to some request received by the vehicle. A report can be of several types depending of the incoming request. A report will always have a reference to the request message which triggered its processing.
3. An event is a message sent by the vehicle to inform other entities involved in the mission that something relevant occurred which should be taken into consideration. Events can be either alarms or detections. Alarms result from detection by the Fault Management system of a failure or an anomaly in the behaviour of one or more components or subsystems of the vehicle, e.g., a loss of battery level, an increase of the internal temperature, the detection of a leak, etc. An alarm is therefore always related to proprioceptive onboard capabilities. On the other hand, a detection results from the observation of the environment of the vehicle either directly by a sensor (altimeter for instance) or through some processing.
4. A query can be defined as a request sent by the vehicle to other entities participating in a data interchange during a maritime mission (such as the CCS or another autonomous maritime vehicle) in order to obtain certain information of interest (e.g., request for an update of the position of other vehicles involved in the mission) or asking for some processing which cannot be done onboard. A query will be processed through the middleware and will result (with a certain delay) in an answer message.

On the other hand, messages sent by the CCS can be of three different kinds: request, notification and answer.

1. A request is an inquiry message that once interpreted by the vehicle will result in a report. There are two types of inquiries depending on whether they are about the mission or about status data. A mission request results in the assignment to the vehicle of a goal, a high level task or a plan to be executed. Attribution of goals or high level tasks require that the vehicle has onboard planning capabilities. A status request usually asks about an update on some kind of proprioceptive data such as remaining autonomy or estimated position.
2. A notification is a message to inform the vehicle that some new relevant information is available. Notifications may or may not require an action by the vehicle. Therefore, they do not imply an answer and could be even ignored by the vehicle. In order to receive a notification, it is mandatory that the vehicle previously subscribes to this kind of information as the middleware that is being used follows a publish/subscription paradigm. Once a notification is received, the vehicle may need to send and explicit query to retrieve associated content. For instance, if notified that there is an updated seabed map available, the vehicle would need to surface, send a query to receive it via radio frequency communications, analyze it and replan the mission if necessary.
3. An answer is a message sent in response to a query emitted by a vehicle. Answers will always have a reference to the query message which triggered its processing.

**Figure 2:**
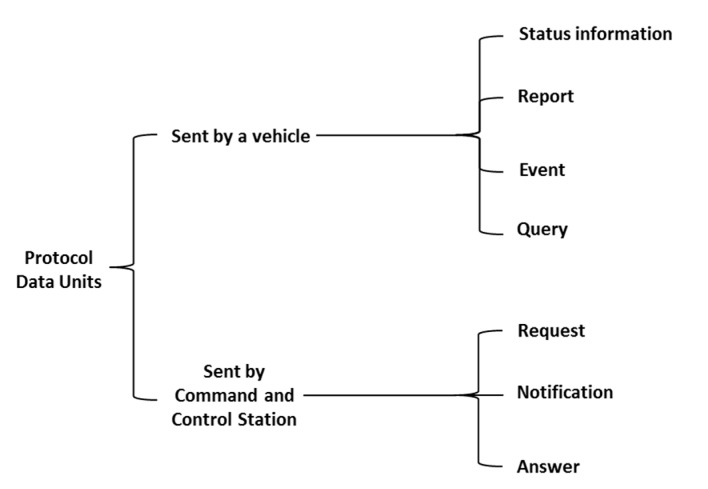
Taxonomy for the PDUs in MDTP.

Thus, there are several possible directions when information involving requests and responses is interchanged among the different devices deployed in a mission: either they are sent from the vehicle to the CCS, from the vehicle to another vehicle and from the CCS to the vehicle itself. Considering these aspects, the taxonomy that has been created for the PDUs in MDTP distinguishes two different kinds of main groups has been represented in Figure 2. The structure that has been followed to create it is related to the two kinds of possible actions that can be taken to transmit and receive a message in a system where all the parties are deployed. In any given situation, messages will be either sent by an autonomous maritime vehicle that is present or by the CCS. When they are sent from the vehicles, they are always related to either a mission or any parameter related to it, such as information about the status of mission where the vehicle is currently involved, a report on the data that is requested, any event that may affect the mission or the vehicle behaviour or a query that the vehicle may have to perform in order to better fulfill a mission. On the contrary, if the message is sent by the CCS they will consist of requests that are done to the robots that have been deployed during the mission, a notification that has to be sent to them in order to modify their behaviour, and an answer that has to be sent to them as a result of a requests that they previously sent. In the end, it can be seen how activities that involving requests and answers for those requests (for messages sent by the middleware) and activities related to notifications about the status of the vehicles (for messages sent from the vehicles).

### 3.3. Messages from the Maritime Data Transfer Protocol

**Figure 3:**
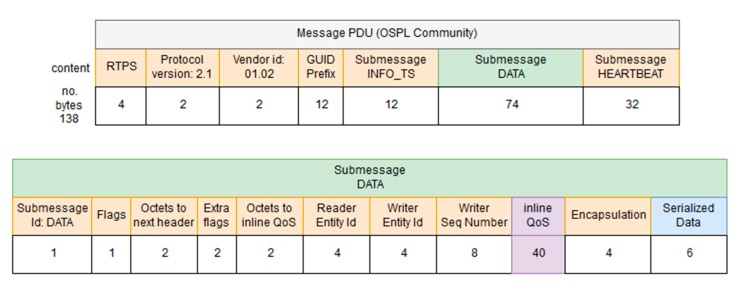
Message PDU with Open Splice DDS Community version.

**Figure 4:**
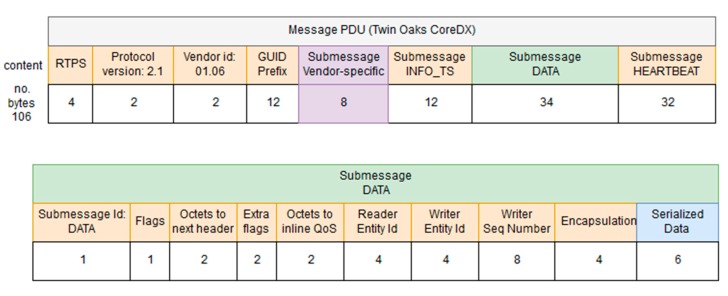
Message PDU with Twin Oaks CoreDX DDS.

The middleware follows the Data Distribution Service (DDS) specification which supports Data-Centric Publish-Subscribe (DCPS) in real-time systems. DDS is a middleware protocol standard for data-centric integration that features extensive fine control of real-time QoS parameters. MDTP relays on the Object Management Group (OMG) standard referred to as DDS Interoperability–Real Time Publish Subscribe wire-protocol (DDSI-RTPS) [42] for implementation works, as it guarantees interoperability with the three main DDS solution providers, i.e., Twin Oaks CoreDX DDS [43], OpenSpliceDDS (PrismTech, Stirling, UK, [44]) and RTI Connext DDS [45]. According to DDSI-RTPS, a message has a header and one to several possible submessages. The header contains information about the RTPS protocol, the vendor and the Globally Unique Identifier (GUID). Each of the submessages has it own submessage header with an id that identifies the type of submessage, and one to several submessage elements. In a communication between the middleware and a vehicle each message PDU will contain three submessages (as represented in Figure 3 and Figure 4): a submessage with information about the timestamp (INFO_TS), a submessage with the data we want to transmit (DATA) and a submessage to inform that there is data available in the writer (HEARTBEAT).

Depending on the vendor, there are differences in the size of the message PDUs. A default PDU with a serialized data of 63 bytes has a size of 138 bytes with Open Splice DDS Community version (OSPL) whereas with Twin Oaks CoreDX DDS, it has a size of 106 bytes. The main difference is that OSPL uses 40 bytes for default QoS specification whereas TwinOaks includes a generic vendor submessage of size 8 bytes. The Serialized Data section of the PDU contains the information to be transmitted between the endpoints of the communication, namely the middleware and AUVs. In our scenario, there are two possible communication channels: an acoustic channel (provided by acoustic modems manufactured by Evologics), to be used when AUVs are underwater, and an IP channel or high bandwidth radio link (e.g., WiFi), to be used when AUVs are at the surface level. Being the bandwidth for acoustic communications very limited, as it has already been explained, the type of information expected to be exchanged between the middleware and AUVs when they are underwater has been analyzed, in order to minimized the size of the PDUs to be transmitted.

Several different kinds of data have been define with the purpose of transferring data according to the needs of a mission, or the tasks it is subdivided in. These different types are as follows:

1. Environment data. These are the data that are related to the surroundings of the location where the autonomous maritime vehicles are deployed.
2. Status: The pieces of information collected from here are about the vehicle that has been deployed. They complement the environment data because status data offer the information that was omitted by them.
3. Situational information: The data deal with the location and movements of the autonomous maritime vehicle that is deployed, such as location, turning parameters or vehicle speed.
4. Other information: These data contain all the information about other features of a deployment, like the algorithm that has been used for positioning.

**Figure 5:**
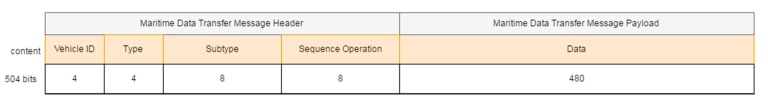
MDTP generic PDU.

The information of interest susceptible to be exchanged has been summarized in Table 3: 

Other types of data bigger in size, like missions, should be transmitted when the AUVs are on the surface so as to use an IP channel with no bandwidth limitations. By missions, it is meant the set of operations that are carried out cooperatively by the autonomous maritime vehicles deployed in order to achieve a complex goal Taking into account the bandwidth restrictions of underwater acoustic communications (low bandwidth, unreliable medium of transmission, difficulties to maintain the nodes in a fixed position, etc.) and the suitability of the time and space decoupling abilities of DDS in those cases, the authors of this manuscript have defined messages for MDTP based on DDS which rely on the previously presented taxonomy of messages (see Section 3.2), and cover all possible scenarios presented by the vehicle providers collaborating in the SWARMs research project (e.g., periodic reports from vehicles, task execution requests, etc.) This protocol enhances the state of the art by providing a definition of the content that should be included in the SerializedData parameter of a DDS submessage of type Data (as shown in Figure 5). The size of the data to exchange through this protocol has been carefully design to adjust to the limited bandwidth of acoustic modems (e.g., an effective bitrate of less than 2 kbps is quite common) to avoid the excessive fragmentation of PDUs in transmissions. This definition is proposed as a standard representation of the information that can be exchanged between the middleware and vehicles in offshore maritime missions. We have defined a size of 63 bytes for this content, comprising a 24 bits header and 480 bits payload:

1. *Vehicle ID (4 bits)*: vehicle identifier of the robot originator or receptor of the message. It has been included in Table 4 and Table 5 as *VID*.
2. *Type (4 bits)*: PDU type. We consider 7 different kinds of PDUs according to the taxonomy of messages defined. Three of them represent the different message types the middleware can send to a vehicle: requests, notifications and answers. The other four represent the message types that vehicles can send to the middleware: periodic status information, reports, events and queries.
3. *Subtype (8 bits)*: PDU subtype. These bits are used to further define the kind of PDU that is sent within the 7 PDU types that have been defined previously.
4. *Sequence Operation (8 bits)*: randomly generated number used to relate petitions and responses.
5. *Data (480 bits)*: 60 bytes available to transfer data. Depending on the message type and subtype, some of these data bytes may be empty. The timestamp associated to this data will be provided by the INFO_TS submessage of the DDS message.

As it was previously mentioned, Figure 3 is containing all the information regarding how PDUs look like in the Open Splice DDS version of DDS. This is an open source iteration of the DDS standard that contains the fields that were mentioned in Section 3.3:

1. RTPS: this field is used to include the RTPS version that is used by the PDUs included in a DDS iteration.
2. Protocol version: used to include the protocol version used in this iteration.
3. Vendor ID: an identifier of the vendor that has developed the DDS iteration.
4. GUID prefix: it is the Globally Unique Identifier used to characterize the message that is sent via DDS.
5. Submessage INFO_TS: contains information about the timestamp of the message that was sent.
6. Submessage data: contains the information transferred.
7. Submessage heartbeat: used to control the periodicity of the information.

At the same time, the PDU that is used for the CoreDX version developed by Twin Oaks has roughly the same fields (as it is an implementation of a standard), but there is an additional one used to include vendor-specific information from the developer. In addition to that, the data submessage is 40 bytes smaller, due to the fact that is not containing the inline QoS information that the OpenSpliceDDS version had. These characteristics have been depicted in Figure 4.

It can be seen that despite claiming that there is interoperability between the Open Splice and CoreDX versions of DDS, both PDUs are of very different characteristics. This is due to the fact that they are different iterations of the DDS standard. Nevertheless, the tests that have been carried out guarantee that information has been transferred with no issues regarding the different features of OpenSpliceDDS and CoreDX. 

### 3.4. Serialized Data

The individual PDUs exchanged both in overwater communications and underwater communications for each of the messages, as classified in the taxonomy proposed in the present paper. Each of the PDUs described is based on the format proposed in Figure 5.

1. *Overwater communications (IP channel)*. As there will not be any bandwidth limitation in this case, the middleware will take advantage of this situation to exchange data with vehicles whose size is expected to be large, like mission plans, that after being defined by the operators of the missions will be transmitted from the middleware to the AUVs before launching them to sea (e.g., if they are on a ship). Table 4 has been used to include all the pieces of information mentioned. In addition to that, the PDUs that are involved in the communications have also been included. They follow the structure that was described, which consists of a vehicle identifier (*VID*), the type of PDU that is used, its subtype (it is required because there are several different kinds of requests, reports or events that can be triggered in a mission), its sequence operation (included as *SeqOp*) and the data used to make the task possible.
2. *Underwater communications (acoustic channel)*. Will make use of PDUs with smaller amounts of data. As it was done in Table 4, the PDUs involved in the communications have also been included. The main reason for MDTP to have been designed is this kind of communication; if only Over-the-air data transmissions existed, it is likely that a different solution would have been studied. However, as described in the open issues, none of the existing options seemed to completely satisfy the required capabilities for data transmissions.

## 4. Implementation and Testing Activities on MDTP

### 4.1. Implementation Works

Once the protocol was fully designed, implementation works were carried out in order to test the usefulness of the solution. A prototype that covers the main features previously described has been implemented. This prototype, which has been used as the platform where MDTP performance was tested, is composed of two parts:

- A Java component that represents *the publish-subscription manager* of the middleware, in charge of the DDS communication with the AUVs. The DDS functionalities of this component are implemented by means of *CoreDX DDS*, a proprietary DDS solution offered by Twin Oaks [43].
- A C++ component that represents *the DDS proxy* of the AUV. This component is composed by two different layers: a communication layer in charge of DDS communication with the middleware, and a translation layer in charge of formatting the information to/from the language or commands supported by the AUV (e.g., ROS). This manuscript focuses on the communication layer, as the purpose of the authors is to validate the PDUs defined under MDTP. The DDS functionalities of the DDS proxy are implemented by means of *Vortex OpenSplice DDS Community Edition* which is an open-source DDS solution offered by PrismTech [44].

The DDS communication between both components has been tested in an acoustic network emulated by means of an online modem emulator provided by Evologics [46], which consists of a simulation of their medium frequency modem S2CR 18/34 whose effective bitrate is 1 kbps. This emulator provides real-time modem working procedures, and implements the same source code and commands that the real acoustic modem does. Besides, as it is accessible remotely via Internet, it allows the simplification of the integration tasks and the minimization of the development costs. Besides, the Evologics emulator is able to simulate specific propagation effects in underwater acoustic communication channels, like signal propagation delays, data packet collision detection, packet synchronization errors, time difference of arrivals on the Ultra-Short Base Line (USBL) grid elements and the movement of a real modem due to sea current. Besides, in order to enable the compatibility of this modem with the DDS protocol, a specific software module called *DDS-acoustic converter* able to implement the translation DDS/Evologics-Acoustic-Framework has been developed in the SWARMs project. This is a C++ component with DDS functionalities implemented by means of Vortex OpenSplice DDS Community Edition.

In order to create a scenario for testing purposes, the *publish-subscription manager* of the middleware, the *DDS proxy* of the vehicle and the *DDS-acoustic converter* were installed on a Linux based Personal Computer. To run the modem emulator, it was also necessary to install the Evologics Dual Media Access Control (DMAC) advanced data-link layer protocol. A specific Interface Description Language (IDL) file has been defined to implement the messages in MDTP. IDL can be defined as a specification language used to describe the data model used in the interface between software components in a language-independent manner. All commercial and open source DDS implementations currently available use an IDL file, also known as Data Definition Language (DDL) file, as the cornerstone for the generation of the data types needed for a specific software implementation. The format of the IDL file that has been defined is the following:

module swarmsIDL
{
struct SWARMsmsg
{
  octet vid_type;      //8 bits (VID bits 7..4, Type bits 3..0)
  octet _subtype;      //8 bits
  octet seqoperation;   //Sequence nr of the operation (8 bits)
  unsigned long dataInt[15];  //15 x 32 bits
 
};
 
};

The first byte represents the Vehicle ID (4 bits) and the Type (4 bits) of MDTP, while the second and the third byte represent the SubType and the Sequence Operation (all of them described in Section 3.3). Finally, 60 bytes have been reserved to transfer data, although the authors of the proposal are aware that not all transmissions will need such an amount of data, so in some cases part of these data bytes may be empty.

### 4.2. Specific PDU Tests

The activities to be carried out to create the scenario where the testing activities are done were as follows: (1) starting the online modem emulator by opening a terminal window and running the Evologics DMAC software. This software automatically accesses to Evologics’ Virtual Private Network (VPN) and starts the online modem emulator; (2) starting the publish-subscription manager of the middleware by opening another terminal window and running this specific piece of software; (3) starting the DDS proxy of the AUV in a new terminal window; (4) Finally, starting the DDS-acoustic converter of the modem emulator in a separate terminal window.

As soon as the DDS components (the DDS-acoustic converter, the publish-subscription manager and the DDS proxy) are started, the auto-discovery of the DDS nodes takes place. The automatic discovery of entities provided by DDS is a useful feature that allows applications to publish and subscribe to data without needing to configure the specific endpoints that they use to interchange information, regardless of whether these nodes are on the same machine or distributed in a network. Each DDS application performs the standard automatic discovery process which includes announcing the presence of its DDS Entities, listening for other DDS Entities, and looking for matches between its own DDS Entities and those discovered. This process usually takes a few seconds only, but due to the especial characteristics of the DDS entities of our implementation based on two different DDS libraries (i.e., the publish-subscription manager is CoreDX DDS based, while the DDS-acoustic converter and the DDS proxy are Vortex OpenSplice DDS Community Edition based), the auto-discovery process takes around 25 s. It must be kept mind that this process shall only be executed once (the moment the scenario is initialized). 

In order to test the developed implementation, three main different cases have been defined: (1) the case where the middleware subscribes to periodic environmental information sent by a vehicle; (2) the case where the middleware requests to a vehicle the execution of a specific task and (3) the discovery of the Control Data Terminal (CDT) component used to know the active vehicles in a deployment. In addition to that, other testing activities that have been carried out involve the case where the middleware subscribes to periodic status information sent by a vehicle.

#### 4.2.1. Test Case 1: Subscription to Periodic Environmental Data

**Figure 6:**
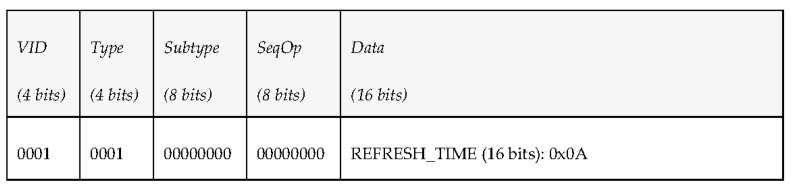
Appearance of the PDU used to request environment information.

The middleware architecture wants to subscribe to periodic environmental information sent by a vehicle, so it publishes a PDU under the topic “request_environment” to request the vehicle the delivery of environment-related data every 10 s. Figure 6 shows the kind of data that were added to the fields that conform the PDU send to do the request.

**Figure 7:**
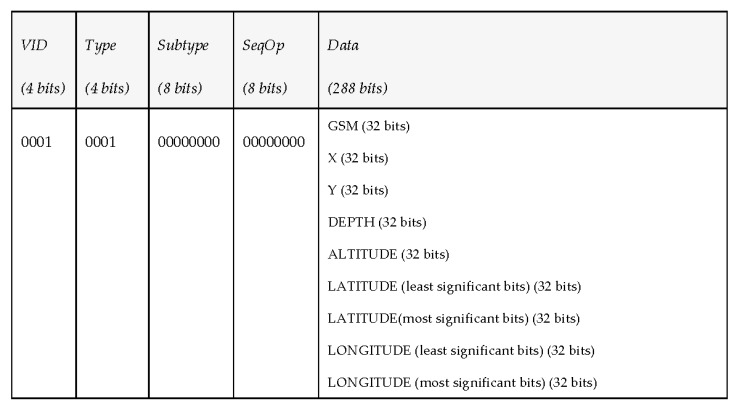
Appearance of the PDU used to send environment information.

Since the vehicle is subscribed to the topic “request_environment”, it processes the petition, takes the correspondent measurements and publishes every 10 s a state vector (under the topic “report_environment”) with the corresponding data shown in Figure 7.

**Figure 8:**
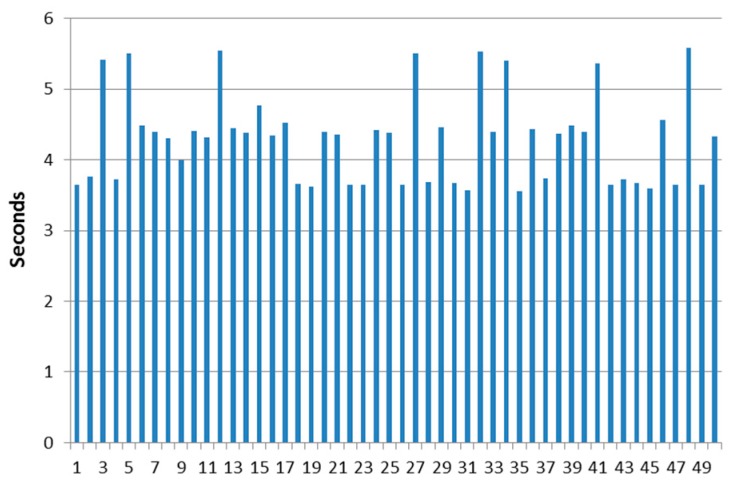
Time measurements for periodic environmental data subscriptions.

The time that it takes for the DDS proxy located in the AUV to answer the requests from the publish-subscription manager of the middleware and send a state vector has been measured as varying between 3.56 and 5.58 s. It has also been verified that if 10 DDS proxy components are started in 10 different terminal windows (i.e., representing a swarm of 10 AUVs deployed underwater), the request sent by the publish-subscription manager reaches the 10 components almost simultaneously. The reliability of the protocol to work among the different parts of the components was tested by means of a series of tests where the PDUs where sent through all the software components used for their interaction. Figure 8 displays the results obtained when running 50 attempts at sending environmental data related to subscriptions.

In this figure, it is shown how the period of time used to transfer data regarding subscriptions can be done in a reliable (there were no connection errors in the scenario that was used) and realistic manner. The data were transmitted at rates that are similar to the ones used in other solutions that involve message transmission in middleware environments: for example, user experience with data transmission in Oracle Fusion Middleware is expected to be measured in seconds (“For example, you might want to ensure that 90% of the users experience response times no greater than 5 s and the maximum response time for all users is 20 s” [47]. Timeout and keep alive messages use time intervals of 300 and 5 s, respectively). JBoss Enterprise Application Platform uses 30 s intervals for timeout messages too [48]. Lastly, it is consistent with results that can be expected from Message-Oriented Middleware, where according to Korhonen, “Online messaging is in real time, with message delivery typically occurring in seconds or even sub-seconds” [49]. Overall, it can be said that this protocol that has been conceived for autonomous maritime vehicles shows a comparable performance with some other already established solutions. Nevertheless, MDTP has been explicitly conceived for transmission of maritime data using as little amount of bits as possible, unlike all the other solutions that make use of a reliable transmission medium.

Table 6 shows that the performance of the system is satisfactory overall: clearly, the amount of time employed to deliver data from a subscription varies within acceptable ranges of time if the data transmission between vehicles is considered. Furthermore, the average and median values obtained from the 50 successful attempts to establish communications are very close one to the other; this implies that the outlier values have little weight in the measurements obtained, and therefore confirms that the protocol offers a predictable performance. This is reinforced by the comparatively reduced time difference between the minimum and maximum figures (minimum is slightly less than 64% of the maximum) that have been measured.

#### 4.2.2. Testing Case 2: Task Execution Request

**Figure 9:**
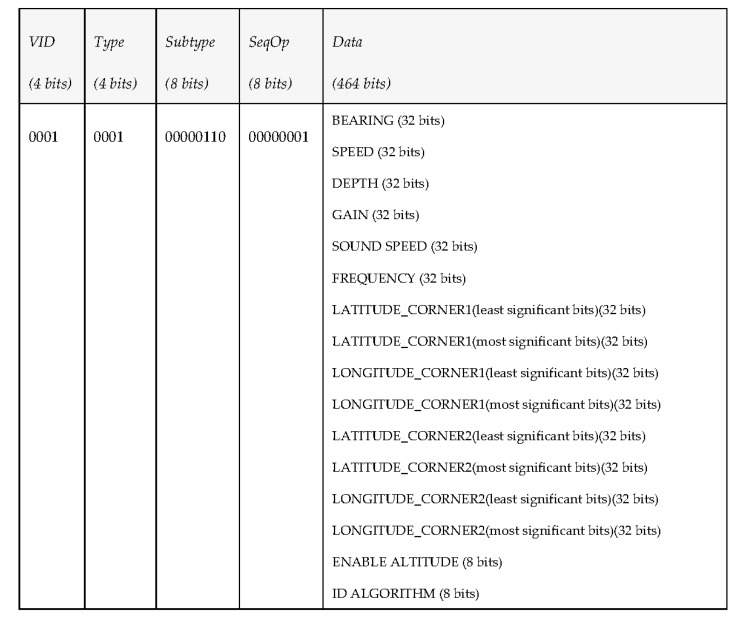
Appearance of the PDU used for area cover request.

In this case, the middleware wants the vehicle to cover an area, so it publishes a PDU under the topic “request task” specifying the needed data to perform such a task. As it can be seen in Figure 9, the information that will be transferred this time will be more abundant than before, due to the fact that it has to include the parameters used to perform the task (thus, since it implies covering an area, the data implies information about how to maneuver the vehicle and area boundaries).

**Figure 10:**
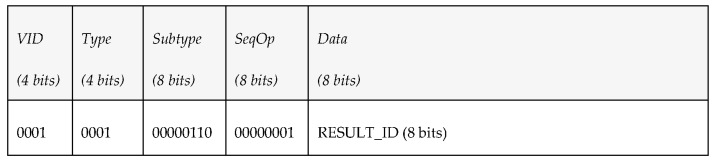
Appearance of the PDU used as an answer for the task reported.

Since it is assumed that the vehicle is subscribed to the topic “request_task”, it processes the petition and publishes the reports of such a task (under the topic “report_task”) as soon as these reports are available. Figure 10 depicts the results of those reports. It can be noted how there is only one field used now, which will be used to communicate whether the request was successful or not.

**Figure 11:**
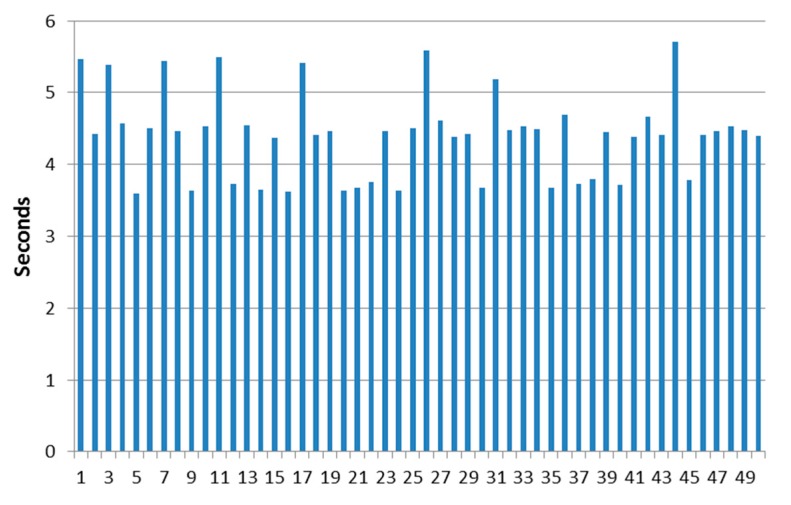
Time measurements for task executions.

The time that it takes the DDS proxy of the AUV to report about the execution of the task demanded by the publish-subscription manager of the middleware varies between 3.6 and 5.71 s. In order to ensure the kind of performance that could be obtained in case there were several task execution requests from a collection of robots deployed in a system rather than just one, 50 more requests attempts were made. As it happened with the previous use case, the range of times obtained (even for outlier samples) was deemed as satisfactory in the simulations where the different components were running under the PDUs described in this protocol. The results of this set of tests have been portrayed in Figure 11. Overall, the performance resembles what was obtained in the previous used case with a small increment in the time required for the requests to be attended, which is to be expected due to the fact that one of the PDUs used for information interchanges is longer than the ones that were used before.

An analysis of the most prominent data that has been obtained and summarized in Table 7 shows similar remarks as the ones that were described in the previous example: maximum and minimum values are included within ranges that are reasonable (minimum value is 63% of the maximum value, which is roughly the same result that was obtained in the previous experiment) and the average and median figures are very close one from each other (also demonstrated by the low value of standard deviation, which shows low dispersion among the values retrieved while fulfilling requests).

#### 4.2.3. Testing Case 3: Discovery of Control Data Terminal Component

**Figure 12:**
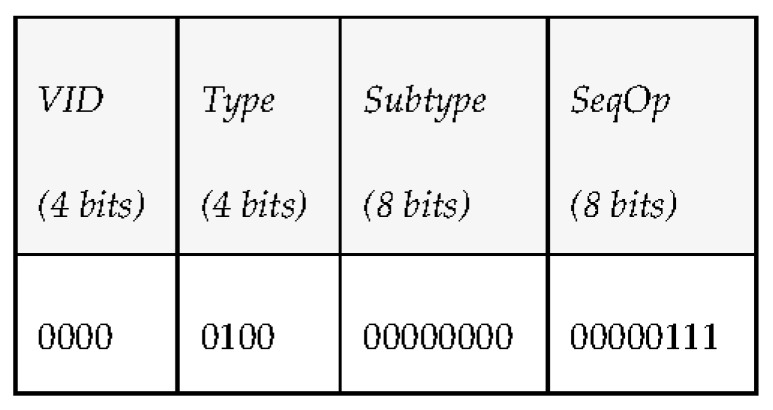
PDU used for CDT discovery.

This use case was introduced with the idea of having an accurate idea of the amount of time required to check the existence of available vehicles present in a deployment. The system where MDTP works is based on the usage of CDTs in order to know which autonomous maritime vehicles are present (since an active CDT will mean that a vehicle has been deployed). While the PDU required to make this possible is simpler than the others, as it can be seen in Figure 12, MDTP has been conceived to work with two different versions of DDS when a system is deployed. Consequently, there are several parameters that must be negotiated and will take a longer time to have that process completed. Fortunately, it is a procedure that will only have to be done once during a mission, so it will not be time consuming or disruptive from the bandwidth usage point of view.

**Figure 13:**
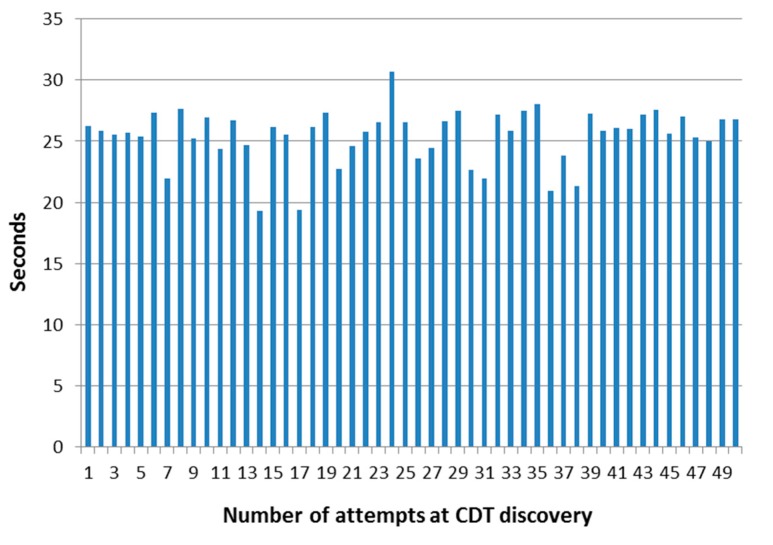
Time measurements for CDT discovery.

The measurements involving a collection of 50 attempts to successfully prove this functionality have been included here too. These are larger than the ones obtained previously due to the negotiations that have to be done regarding the different DDS versions used in the designed system, but discovery is carried out successfully in any case. Furthermore, no discovery requests or interchanged data were lost during the testing activities. These features can be appreciated in Figure 13, where the results are shown in a graphical manner. 

With regards to the most prominent figures obtained from the set of run tests, despite been higher than the ones obtained previously (due to the parameters that must be negotiated between the two different implementations of DDS) they still show the same positive features that were shown before. For example, the difference between the minimum and maximum time figures is still contained (the minimum value is 62.9% of the maximum). What is more, as it can be seen in Table 8, the difference between average (25.4436 s) and median (25.885 s) parameters is small enough to confirm the stability of the protocol under the scenario that has been put forward.

#### 4.2.4. Other Testing Activities

**Figure 14:**
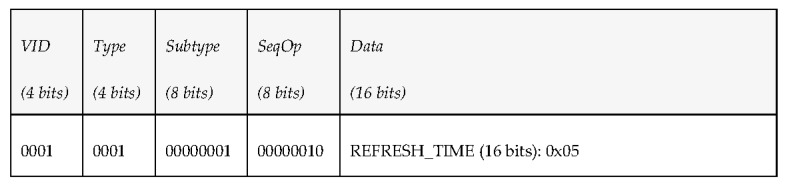
Appearance of the PDU used as a request for periodic mission status data.

Among the other functionalities conceived to make use of MDTP for data transmissions, creating a subscription to periodic mission status data is one of the most important. In this case, the middleware wants to subscribe to the periodic mission status data sent by a vehicle, so it publishes a PDU under the topic “request_mission” to request the vehicle to send the mission status every 5 s. The overall structure of the PDU, as depicted in Figure 14, is oriented to information requests.

**Figure 15:**
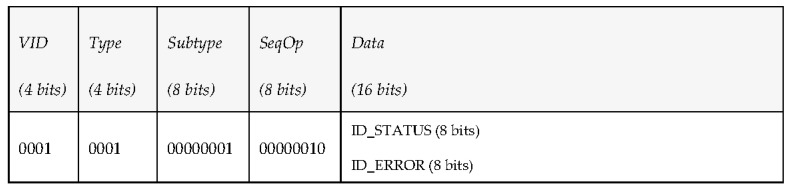
Appearance of the PDU used as an answer for periodic mission status data.

As the vehicle is subscribed to the topic “request_mission”, it processes the petition and publishes a mission state message (under the topic “report_mission”) with the corresponding data every 5 s. The answer will provide an identifier with the status of the mission and another identifier for the error. Its overall appearance is displayed in Figure 15.

The time that it takes the DDS proxy of the AUV to answer the request from the publish-subscription manager of the middleware and send the mission status was measured as varying between 3.75 and 4.60 s. As in test case 1, if several DDS proxy components were started, the request send by the publish-subscription manager reaches all these components almost simultaneously. Interestingly enough, the time required to make the data delivery is only slightly higher than the one required in the first test. Most of the information sent in this test is contained in the DDS header, which is mandatory to correctly implement the defined publish-subscribe mechanism and apply the QoS settings.

### 4.3. Discussion of the Development Work

As it has been shown, the undertaken development works show the feasibility of the protocol that has been put forward. This version of the protocol includes more complex features that have been codified for the integration stages of the SWARMs project, which constitutes the framework used for the development of MDTP. Even with them, the performance of the protocol regarding transmission of information at the data level has been proven satisfactory, as data are transmitted at a pace acceptable by both the deployed hardware and any potential human operator. Even when more complex information is transmitted, such as GPS coordinates, the protocol will handle the data without issues or errors and will be transmitted and understood correctly at both ends of the communication. In addition to these, there are some other features that have a major importance for the development works that have been taken into account for the deployment of the protocol:

1. A nominal bitrate of 1 kbps is quite common for underwater communications. While limitations on the bandwidth for underwater communications have been explained in this manuscript, it must be taken into account that 1 kbit per second data transmission are the usual ones present in acoustic communications. It is because of this reason that PDUs cannot be larger than a certain limit of bytes, or otherwise data will be lost. At the same time, trying to send information in single PDUs that do not relay on other ones to provide all the information requested is also something to consider, as such a procedure will maximize the chances of having all the data correctly delivered to the destination.
2. The simulator that has been used to mimic the behaviour of underwater communications in the open sea is based on what is used in the SWARMs project, that is to say, the model S2C18/34 manufactured by Evologics (Berlin, Germany), which matches the required performance for transmission of data over relatively large distances (up to 3.5 km) with data rate up to 13.8 kbps (nominally). Although this bitrate will usually fall well below this figure when communications take place in shallow waters (due to reflections in the acoustic waves), it will still be usable to transmit information.
3. If one acoustic modem is responsible to transmit the periodic data sent by several AUVs, the period for the transmission of periodic data will be no lower than 10 or 15 s in order to have the acoustic modem working correctly. This is due to the fact that, due to the characteristics of the environment where data transmissions take place, it is not advisable to establish periodic data transfers at a rate of milliseconds, especially if there are several vehicles transmitting information at the same time, because they might overall the modem, regardless of the strategy used to send that information (for example, if too many PDUs gather at a point, the modem will give priority to the first data received, in a First-In-First-Out fashion).

## 5. Conclusions and Future Works

A study of the existing protocols capable of offering compelling enough features, while at the same time being used in the environment that has been described (underwater autonomous maritime vehicles, low bandwidth availability, unreliability for transmissions at the data level), has been included in Section 2. The open issues and challenges that have been found are dealt with by the protocol that has been thoroughly described in Section 3. Since the protocol is expected to be more than just a mere design, implementation works have been carried out, as described in Section 4. As far as the authors are concerned, a protocol tailored for the needs of transmissions at the data level among distributed elements of a collection of maritime vehicles is not presented in any piece of literature. Furthermore, its usefulness is guaranteed, as it is expected from it that will transfer information in a level of detail and accuracy that is hardly matched by any other current proposal.

There are several future works to be undertaken, as part of the activities to be done in the SWARMs project. The protocol will be tested with the plethora of vehicles expected to be used in the project, which range from AUVs to ASVs or ROVs. Their differences in performance and computational capabilities should not be significant for the protocol, as it is lightweight enough to guarantee that it can be used in a transparent way by them. Furthermore, any partial modification that has to be done on the protocol will be tackled, even though after collecting all the information that is required for the application domain where it is used, changes or further extensions of MDTP seem unlikely. Finally, even though there is inter-compatibility among the different versions of DDS, the usage of a single one at both ends of the communication will be taken into account in order to optimize the procedure used for automatic discovery of elements during data transfer. This could provide an important advantage due to the fact that using a single DDS distribution will minimize the amount of time required for automatic discovery, as it has been defined in the standard.

## Figures and Tables

**Table 1:** Summarized advantages and disadvantages of the studied proposals.

Name of the Proposal	Advantages	Disadvantages	References
Deadline-Constrained 802.11 MAC	Accurate definition of information transfers in the transmission medium.	Works at a lower level (physical and network access layers rather than middleware or data-based ones).	Tian, G., S. Camtepe, and Y.C. Tian, *A Deadline-Constrained 802.11 MAC Protocol With QoS Differentiation for Soft Real-Time Control* [21].
Constrained Application Protocol	PDU definition and interchange are thoroughly described. It has been designed for constrained environments.	Conceived for the application layer rather than a level below.	Internet Engineering Task Force (IETF), *The Constrained Application Protocol, RFC 7252 (CoAP)* [22].
Advanced Message Queuing Protocol	Set of PDUs for information transmission at the data level. It has a broker for Publish/Subscribe communications.	Conceived for reliable transmission mediums rather than an underwater channel. Adding security can be challenging.	AMQP consortium. *AMQP 1.0 Becomes OASIS Standard* [24].
MACA-based power control	Reliable performance in underwater environments where bits are transmitted.	Focused on bit transmission, rather than data level with higher level information.	L. Qian, S. Zhang, M. Liu and Q. Zhang. *A MACA-based power control MAC protocol for Underwater Wireless Sensor Networks* [27].
Power-efficient routing protocol	Optimized scheme for bit transmission.	It is not a suitable solution for transmissions at the data level.	Chenn-Jung Huang, Yu-Wu Wang, Hsiu-Hui Liao, Chin-Fa Lin, Kai-Wen Hu, Tun-Yu Chang. *A power-efficient routing protocol for underwater wireless sensor networks* [28].
Message Queue Telemetry Transport	Solution suitable for distributed systems.	This proposal has not been conceived for underwater environments.	MQTT consortium. *MQTT Version 3.1.1* [29]. Locke, D., *Mq telemetry transport (mqtt) v3.1 protocol specification* [30]*.*
MQTT-SN	Includes Publish/Subscribe communications.	It has been developed for sensor networks instead of underwater environments.	Andy Stanford-Clark, H.L.T., *MQTT For Sensor Networks (MQTT-SN) Protocol Specification* [33].
Embedded binary HTTP	Small-sized transmissions with a similar approach to CoAP.	Solution conceived for the application layer rather than lower ones. Power demanded may be too much for constrained devices.	Tolle, G., *Embedded Binary HTTP (EBHTTP)* [34].
Extensible Messaging and Presence Protocol	Enables security. Follows a CoAP approach.	Lack of Quality of Service capabilities. XML might be troublesome for underwater data transmissions.	Saint-Andre P (technical representative of the Internet Engineering Task Force, *Extensible messaging and presence protocol (xmpp)* [35].
Lightweight M2M	Management of a wide range of embedded systems.	It is built on top of CoAP, so it is unsuitable for underwater data transmissions.	Tian L. OMA device management working group (OMA DM WG), *Lightweight m2m (oma lwm2m)* [38].

**Table 2:** Actions taken by MDTP.

Name of the Proposal	Disadvantages	Action Taken in MDTP to Deal with the Disadvantage
Deadline-Constrained 802.11 MAC	Works at a lower level (physical and network access layers rather than middleware or data-based ones).	MDTP is used at the data level for data-based information transfers.
Constrained Application Protocol	Conceived for the application layer rather than a level below.	Used at the data level (as PDUs used by a middleware solution) rather than one level above (application) or levels below (transport, network).
Advanced Message Queuing Protocol	Conceived for reliable transmission mediums rather than an underwater channel. Adding security can be challenging.	Designed for underwater transmissions (the ones deemed as more difficult) from scratch. Security is also offered as an option.
MACA-based power control	Focused on bit transmission, rather than data level with higher level information.	Used at the data level (as PDUs used by a middleware solution) rather than one level above (application) or levels below (transport, network).
Power-efficient routing protocol	It is not a suitable solution for transmissions at the data level.	Used at the data level (as PDUs used by a middleware solution) rather than one level above (application) or levels below (transport, network).
Message Queue Telemetry Transport	This proposal has not been conceived for underwater environments.	Designed for underwater transmissions (constrains and unreliability of the transmission medium are taken into account).
MQTT-SN	It has been developed for sensor networks instead of underwater environments.	Designed for underwater transmissions (constrains and unreliability of the transmission medium are taken into account).
Embedded binary HTTP	Solution conceived for the application layer rather than lower ones. Power demanded may be too much for constrained devices. It relies on a layered protocol architecture likely not to be present in an underwater environment.	MDPT can be used on top of regular IP networks or separately over underwater, acoustic-based deployments.
Extensible Messaging and Presence Protocol	Lack of Quality of Service capabilities. XML might be troublesome for underwater data transmissions.	Non-verbose format is used for data transmissions. QoS is guaranteed by its inclusion of MDTP in a DDS development.
Lightweight M2M	It is built on top of CoAP, so it is unsuitable for underwater data transmissions.	Designed for underwater transmissions (constrains and unreliability of the transmission medium are taken into account).

**Table 3:** Information to exchange when AUVs are underwater.

Data	Units	Size	Data Type	Range of Values
**Environment data**				
Water temperature	Celsius	8 bits	Byte	−3.75–60.5 °C
Water salinity	Parts per million	16 bits	Unsigned short	0–65534 ppm
Sound Velocity	m/s	32 bits	Float	0.0–9999.999 m/s
Turbidity	Parts per million	16 bits	Unsigned short	0–65534 ppm
Pollution (H2S)	Parts per million	16 bits	Unsigned short	0–65534 ppm
Currents	cm/s	8 bits	Unsigned byte	0–254 cm/s
Bathymetry–underwater maps (sonar)	m	32 bits	Float	0.0–9999.999 m
**Status**				
Vehicle battery	%	8 bits	Unsigned byte	0.0–100.0%
Temporal reference	s	16 bits	Unsigned byte	0–65534 s
Sensor status	Bit identifier + bit mode	8 bits (5 bits + 3 bits)	Unsigned short	0–31 sensors, 0–8 modes
Vehicle event/alarm	Event or alarm code	4 bits	Byte	0000–1110 (15 different events/alarms)
**Situational information**				
Latitude	Degrees	64 bits	Float	−90–90
Longitude	Degrees	64 bits	Float	−180 to 180
Coordinates (Inertial/USBL/DVL)	cm/s	8 bits	Unsigned byte	0–254 cm/s
Working depth	m	32 bits	Float	0–4095.875 m
Euler Angles (Pitch)	Degrees	32 bits	Float	0.000–360.000°
Euler Angles (Roll)	Degrees	32 bits	Float	0.000–360.000°
Euler Angles (Yaw)	Degrees	32 bits	Float	0.000–360.000°
Distance to seabed	m	16 bits	Unsigned short	0–4095.875 m
Azimuth	Degrees	32 bits	Float	0.000–360.000°
Vehicle speed	m/s	32 bits	Float	0.0–127.99609375 m/s
Bearing	Rad	32 bits	Float	[−π, π]
Gain		32 bits	Float	
Altitude	m	8 bits	Float	
GSM	m/s	32 bits	Float	
X	m	32 bits	Float	
Y	m	32 bits	Float	
Target Yaw	Degrees	32 bits	Float	0.000–360.000°
Target depth	m	16 bits	Unsigned short	0–4095.875 m
**Others**				
Algorithm Identifier		8 bits	Octet	

**Table 4:** Serialized data for overwater communications.

Message Type	Actions Involved	PDUs Involved				
***Middleware Requests***						
*New Task of a Mission*	The middleware sends a **request** message with a new TASK of a mission. Tasks are defined by the subtype that is included in them. To define the mission, the middleware will send several PDUs like this one to specify the different tasks to accomplish. For example, the task “Go to take picture” is defined by the subtype “00000100”, task “hover to take a picture” by the subtype “00000101”, “cover area” by “00000110”, etc.	*VID (4 bits)*	*Type (4 bits)*	*Subtype (8 bits)*	*SeqOp (8 bits)*	*Data (60 bytes)*
		ID	0001	00000100	NUM	TASK
	The vehicle **reports** to the middleware whether the task was successfully registered (ACK = 00) or not (ERROR_ID).	*VID (4 bits)*	*Type (4 bits*	*Subtype (8 bits)*	*SeqOp (8 bits)*	*Data (1 byte)*
		ID	0001	00000100	NUM	ACK

**Table 5:** Serialized data for underwater communications.

Message Type	Actions Involved	PDUs Involved				
***Vehicle Periodic Messages***						
*Periodic Status Information*	The middleware sends a **request** message to subscribe to periodic data of a vehicle, specifying the refresh time desired in seconds (REF_TIME).	*VID (4 bits)*	*Type (4 bits)*	*Subtype (8 bits)*	*SeqOp (8 bits)*	*Data (2 bytes)*
		ID	0001	00000000	NUM	REF_TIME
	The vehicle **periodically reports** about the kind of DATA that the middleware has subscribed to.	*VID (4 bits)*	*Type (4 bits)*	*Subtype (8 bits)*	*SeqOp (8 bits)*	*Data (60 bytes)*
		ID	0001	00000000	NUM	PDATA
***Middleware Requests***						
*Task Update*	The middleware sends a **request** message with a new task (UTASK) of a mission. Tasks are as defined previously, and will be identified by the same kinds of subtypes.	*VID (4 bits)*	*Type (4 bits)*	*Subtype (8 bits)*	*SeqOp (8 bits)*	*Data (60 bytes)*
		ID	0001	10000010	NUM	UTASK
	The vehicle **reports** to the middleware whether the task was successfully registered (ACK = 00) or not (ERROR_ID).	*VID (4 bits)*	*Type (4 bits)*	*Subtype (8 bits)*	*SeqOp (8 bits)*	*Data (1 byte)*
		ID	0001	10000010	NUM	ACK
*Configure Camera*	The middleware **request** the vehicle to change the configuration of the camera.	*VID (4 bits)*	*Type (4 bits)*	*Subtype (8 bits)*	*SeqOp (8 bits)*	*Data (60 bytes)*
		ID	0001	10000001	NUM	IMACON
	The vehicle **reports** to the middleware whether the camera configuration was successfully done (ACK = 00) or not (ERROR_ID).	*VID (4 bits)*	*Type (4 bits)*	*Subtype (8 bits)*	*SeqOp (8 bits)*	*Data (1 byte)*
		ID	0001	10000001	NUM	ACK
***Vehicle Events***						
*Vehicle Alarms*	The middleware **subscribes** to the alarms of the vehicle.	*VID (4 bits)*	*Type (4 bits)*	*Subtype (8 bits)*	*SeqOp (8 bits)*	*Data (60 bytes)*
		ID	0010	00000000	NUM	SALARM
	The vehicle sends information about the **ALARMs** the middleware is subscribed to.	*VID (4 bits)*	*Type (4 bits)*	*Subtype (8 bits)*	*SeqOp (8 bits)*	*Data (60 bytes)*
		ID	0010	00000000	NUM	ALARM
*Vehicle Detections*	The middleware **subscribes** to the detections of the vehicle.	*VID (4 bits)*	*Type (4 bits)*	*Subtype (8 bits)*	*SeqOp (8 bits)*	*Data (60 bytes)*
		*ID*	*0010*	*00000001*	*NUM*	*SDETEC*
	The vehicle sends the middleware information about some **detection**.	*VID (4 bits)*	*Type (4 bits)*	*Subtype (8 bits)*	*SeqOp (8 bits)*	*Data (60 bytes)*
		ID	0010	00000001	NUM	DETECT
***Middleware Notifications***						
*Middleware Notifications*	The vehicle subscribes to **notifications** from the middleware.	*VID (4 bits)*	*Type (4 bits)*	*Subtype (8 bits)*	*SeqOp (8 bits)*	*Data (60 bytes)*
		ID	0010	00000010	NUM	SNOTIFY
	The middleware sends a **notification.**	*VID (4 bits)*	*Type (4 bits)*	*Subtype (8 bits)*	*SeqOp (8 bits)*	*Data (60 bytes)*
		ID	0010	00000010	NUM	NOTIFY
***Vehicle Queries***						
*Vehicle Queries*	The vehicle **queries** the middleware.	*VID (4 bits)*	*Type (4 bits)*	*Subtype (8 bits)*	*SeqOp (8 bits)*	*Data (60 bytes)*
		ID	0011	00000000	NUM	QUERY
	The middleware **answers** the vehicle.	*VID (4 bits)*	*Type (4 bits)*	*Subtype (8 bits)*	*SeqOp (8 bits)*	*Data (60 bytes)*
		ID	0011	00000000	NUM	ANSWER

**Table 6:** Most prominent figures obtained from periodic data subscription measures

Request Attempt	Time (s)
Maximum	5.58
Minimum	3.56
Average	4.2936
Median	4.365
Standard deviation	0.63554174

**Table 7:** Most prominent figures obtained from periodic data subscription measures.

Request Attempt	Time (s)
Maximum	5.71
Minimum	3.6
Average	4.4002
Median	4.455
Standard deviation	0.59223027

**Table 8:** Most prominent figures obtained from CDT discovery measures.

Request Attempt	Time (s)
Maximum	30.67
Minimum	19.28
Average	25.4436
Median	25.855
Standard deviation	2.24441871
